# Steam Explosion Treatment of Byproduct Feedstuffs for Potential Use as Ruminant Feed

**DOI:** 10.3390/ani9090688

**Published:** 2019-09-16

**Authors:** Yue Liu, Xiaoxuan Ren, Hao Wu, Qingxiang Meng, Zhenming Zhou

**Affiliations:** State Key Laboratory of Animal Nutrition, College of Animal Science and Technology, China Agricultural University, Beijing 100193, China

**Keywords:** steam explosion, byproduct feedstuff, chemical composition, *in vitro* digestibility, energy value, CNCPS composition

## Abstract

**Simple Summary:**

Feedstuffs, such as cassava alcohol residue or potato starchy residue, have not been traditionally used as ruminant feeds for commercial production due to high fiber content and low digestibility. Steam explosion treatment can break down lignocellulosic structural components with no (or few) chemical agents, is environmentally friendly, and uses little energy, which offers great potential for large-scale application. We studied the effect of steam explosion treatment on the chemical composition, *in vitro* digestibility, energy value, Cornell Net Carbohydrate and Protein System (CNCPS) composition of five byproduct feedstuffs, to provide a theoretical basis for the development and utilization of the steam explosion technique to produce ruminant feedstuff. The data revealed that steam explosion treatment improved the nutritive value and *in vitro* dry matter digestibility of cassava alcohol residues, distillers’ grains, rapeseed meal and potato starchy residue. Therefore, steam explosion treatment offers the potential to improve the suitability of these feedstuffs.

**Abstract:**

Although many byproducts of milling industries have potential as a ruminant feed, they have not been widely used due to their low nutritive value, especially high-fiber content and difficult processing techniques. Steam explosion can increase the degradation of hemicellulose, cellulose and lignin and make byproduct feedstuffs more suitable as ruminant feed. Five byproduct feedstuffs: cassava alcohol residue (CAR), distillers’ grains (DG), cottonseed meal (CM), rapeseed meal (RM) and potato starchy residues (PSR), were steam-exploded using five different processing parameters and the effects on the chemical composition, *in vitro* digestibility, energy value, and Cornell Net Carbohydrate and Protein System composition were assessed in order to provide a theoretical basis for the technique’s development and utilization for ruminant feed production. In this study, after steam-explosion treatment, the nutritive value and *in vitro* dry matter digestibility (IVDMD) of CAR, DG, RM and PSR were improved (*p* < 0.05), while there was no effect on nutritive value of CM (*p* > 0.05). Specifically, steam explosion treatment decreased the contents of neutral detergent fiber, acid detergent fiber, available cell wall, and slowly degraded protein, and increased the total digestible nutrients, digestible energy, metabolic energy, net energy for maintenance, and net energy for gain, sugar, non-structural carbohydrate and IVDMD. Therefore, steam-explosion treatment offers the potential to improve the suitability of byproduct feedstuffs as ruminant feed.

## 1. Introduction

In recent years, a growing global population and changing consumption patterns require sustainable food production and increasing amounts of animal proteins [1]. Large quantities of global agricultural and food-industry residues such as those from the oil-pressing industry, sugar industry and food processing account for about 30% of agricultural production [2]. These residues can lead to vast waste of biological resources and cause environmental pollution if they are not handled properly [3]. These residues have the potential to become feed with a high nutritive value and to produce more animal protein for human consumption, if fully utilized [2]. It is of great interest to improve the nutritive value of byproduct feedstuffs [4], which have not been traditionally fed to animals or sold as commercial feed [2]. The rumen of ruminants contains a large variety and number of microorganisms, including bacteria, fungi, protozoa and archaea, which ferment low-quality, high-fiber feed to produce volatile fatty acids and microbial proteins to provide energy [5]. Therefore, ruminants are important for sustainable food systems, by converting human inedible byproduct feedstuffs such as rice bran, distillery by-products, cottonseed meal or crop stover [2] into milk and meat for human consumption through digestion by rumen microorganisms. However, byproduct feedstuffs with a low nutritive value and high fiber content might impair the stability and function of rumen microorganisms [5] and have not been widely used in production. Methods for improving the nutritive value of byproduct materials have been reported, including chemical [6], biology [7], and physical simple treatment [8]. Among these, physical methods present many advantages, for example, low cost, short time, simple treatment, and environmental friendliness.

Steam explosion is a thermo-mechanicochemical pretreatment technique that degrades lignocellulosic structural components through heating, organic-acid formation, and shearing forces caused by the explosive expansion of intrinsic moisture during pressure release [9]. The greatest advantage of steam explosion pretreatment is the low energy consumption, Holtzapple et al. [10] compared the explosive depressurization method and conventional mechanical method in reducing the size of poplar and aspen wood, the former requiring about 40% less energy than the latter. Furthermore, steam explosion is environmentally friendly, since no (or very few) chemical agents are necessary [9], and it offers greater potential for large-scale application. The application of steam-explosion treatment in converting byproduct feedstuff into ruminant feed has not been studied. Therefore, the objective of this study investigates the effects of steam-explosion treatment on the nutritive value and *in vitro* digestibility of five byproduct feedstuffs and provides a scientific basis for the feasibility of the development and application of the steam-explosion technique to a variety of byproduct feedstuffs.

## 2. Materials and Methods 

### 2.1. Raw Materials and Steam-Explosion Treatment

Five byproduct feedstuffs: cassava alcohol residue (CAR), distillers´ grains (DG), cottonseed meal (CM), rapeseed meal (RM) and potato starchy residue (PSR) were obtained from Hebei, China in August 2018. A subsample was treated using a steam explosion unit (China Agricultural University, Beijing, China). According to the different characteristics of each feedstuff, different steam-explosion processing parameters were selected, as follows: CAR—0.3 Mpa, 1 min; 0.8 Mpa, 0 s: DG—0.6 Mpa, 10 s; 1.7 Mpa, 30 s: CM—0.3 Mpa, 30 s; 0.8 Mpa, 0 s: RM—0.3 Mpa, 1 min; 0.8 Mpa, 0 s: PSR—0.3 Mpa, 3 min; 0.5 Mpa, 0 s. The first parameter represents the pressure of the 800-L sealed tank after it was filled with high-pressure steam, the second is the duration at which this pressure was maintained, the third parameter is the pressure to which the tank was increased, before pressure was released, and the last is also the duration at which this pressure was maintained. Three replicate experiments were performed for each feedstuff and each experiment with each feedstuff requires 100 kg raw material. Raw and steam-exploded samples were air-dried at 65 °C for 48 h (DHG-9070A, Shanghai hualian medical equipment co., LTD, Shanghai, China), then ground to pass a 2-mm sieve in a feed mill (DF-20, Wenling Linda machinery co. LTD, Taizhou, Zhejiang, China).

### 2.2. Chemical Composition

The determinations of dry matter (DM) (method 930.15) and Ash (method 942.05) were based on those of the AOAC (2000) [11]. The content of nitrogen was determined by the Dumas combustion method (RaPid N III, Elementar Analysensysteme, Garmany), and the contents of crude protein (CP), neutral detergent insoluble protein (NDIP) and the acid detergent insoluble protein (ADIP) were obtained by multiplying the nitrogen by 6.25. The content of ether extract (EE) was analyzed using an automatic extractor (ANKOM XT101, ANKOM Technology Corporation, Macedon, NY, USA). The amounts of neutral detergent fiber (NDF), acid detergent fiber (ADF), and acid detergent lignin (ADL) were determined using a fiber analyzer (ANKOM A220, ANKOM Technology Corporation, Macedon, NY, USA) with heat-stable-amylase according to Van Soest et al. [12] and Robertson and Van Soest [13].

### 2.3. In Vitro Digestibility

*In vitro* incubation was performed using the procedure described by Menke et al. [14]. Rumen liquid was collected from three Angus steers (450 ± 15 kg) fitted with a permanent rumen fistula (Beef Cattle Research Center, China Agricultural University, Beijing, China). The animals used in this experiment were approved by the China Agricultural University Animal Welfare and Animal Experiment Ethical Review Committee (AW42059102-2, Beijing, China). Animals were fed twice-daily with a total mixed ration according to NRC 2016 [15], and were freely provided with drinking water. Each rumen sample (0.5 g) was placed into a small nylon bag (3.5 × 8 cm), sealed with a heat sealer (FR-300B, Shanghai yiguang packing machinery co., LTD, Shanghai, China), then put into a 100-mL culture tube, then the tubes were pre-warmed to 39 °C (DHG-9070A, LTD). Rumen fluid was collected in a vacuum bottle before the morning feed, then was immediately strained through four layers of cheesecloth. The rumen fluid was mixed with buffer solution in a 1:2 ratio (*v*:*v*) with the continuous injection of CO_2_ at 39 °C, then 80 mL mixed liquor was pipetted with an automatic pump into each culture tube followed by incubation in a water bath at 39 °C for 48 h. At the end of incubation, the nylon bags were immediately immersed in cold water, washed by hand until the water became clear, dried at 105 °C for 12 h, then in vitro dry matter digestibility (IVDMD) was calculated.

### 2.4. Energy Value

The amount of total digestible nutrients (TDN) was calculated according to Weiss [16], and the digestible energy (DE), metabolic energy (ME), net energy for maintenance (NEm) and net energy for gain (NEg) were calculated based on the TDN [15]. Chemical compositions were expressed as % DM, energy values were expressed in Mcal/kg.

TDN (%) = 0.98 × (100 − NDF_N_ − CP − Ash + IADIP − EE) + K_dCP_ × CP + 2.25 × (EE − 1) + 0.75 × (NDF_N_ − ADL) × [1 − (ADL / NDF_N_)^0.667^] − 7

NDF_N_ = NDF − NDIP + IADIP

IADIP = 0.7× ADIP

K_dCP_ = exp (−0.0012 × ADIP)

DE = TDN (%)/4.4

ME = 0.82 × DE

NEm = 1.37 × ME − 0.138 × ME^2^ + 0.0105 × ME^3^ − 1.12

NEg = 1.42 × ME − 0.174 × ME^2^ + 0.0122 × ME^3^ − 1.65

### 2.5. The Cornell Net Carbohydrate and Protein System (CNCPS) Composition

The determinations of soluble crude protein (SCP) and non-crude protein nitrogen (NPN) were performed as reported by Licitra et al. [17]. Starch was analyzed using the total starch assay kit (Megazyme, Bray, Ireland; method 996.11) according to AOAC 2000 [11]. The compositions of carbohydrate (CHO), sugar (CA), starch (CB1), available cell wall (CB2), unavailable cell wall (CC), non-structural carbohydrate (CNSC), and the protein compositions of non-protein nitrogen (PA), rapidly degraded protein (PB1), intermediately degraded protein (PB2), slowly degraded protein (PB3) and bound protein (PC) were calculated according to Sniffen et al. [18]. Chemical compositions were expressed as % DM.

Carbohydrate composition:

CHO = 100 − CP − EE − Ash

CC = 2.4 × ADL

CB2 = NDF − NDIP − CC

CNSC = CHO − CB2 − CC

CB1 = Starch

CA = CNSC − CB1

Protein composition:

PA = NPN

PB1 = SCP − PA

PC = ADIP

PB3 = NDIP − ADIP

PB2 = 100 − PA − PB1 − PB3 − PC

### 2.6. Statistical Analysis

All data in this experiment were analyzed using the repeated measures of general linear model procedure of SPSS (version 20, IBM Corporation, Armonk, New York, NY, USA). Additionally, *p* < 0.05 was considered as a significant difference.

## 3. Results

### 3.1. Chemical Composition and In Vitro Digestibility

Table 1 shows the chemical compositions and in vitro digestibility of raw and steam-exploded samples, mainly including the DM, NDF, ADF, ADL, CP, EE, Ash, and 48 h IVDMD. In general, steam explosion treatment had effect on chemical compositions and 48 h IVDMD (*p* < 0.05), where the contents of NDF in CAR, DG, and RM (*p* < 0.01) decreased by 14.64%, 19.04%, and 1.64%, respectively, and the contents of ADF decreased (ranging from 10.42% (DG) to 1.23% (RM)) (*p* < 0.05), and the contents of ADL in DG and CM also decreased (*p* < 0.05); while, after 48 h, the IVDMD increased by 32.06%, 30.36%, 9.97%, 16.75%, 8.82%, respectively (*p* < 0.01). Furthermore, the contents of CP, EE and Ash decreased and increased to varying degrees.

### 3.2. Energy Value

The energy values data are depicted in Table 2. Steam explosion treatment increased (*p* < 0.05) the TDN and energy values of CAR, DG, RM, and PSR, but not CM (*p* > 0.05). Increased TDN (*p* < 0.05) ranged from high to low were CAR (52.10%), 24.83% (DG) and RM (1.42 %).

### 3.3. The Cornell Net Carbohydrate and Protein System Composition

#### 3.3.1. Carbohydrate Composition

Table 3 shows the CHO, CA, CB1, CB2, CC, and CNSC of carbohydrate compositions. Following treatment, PSR showed the highest increase in CHO (*p* < 0.01), and also increased in CA, and CNSC (*p* < 0.05). Moreover, CA, CC, and CNSC of CAR increased by 92.09%, 21.71%, 81.38%, respectively (*p* < 0.01), as well as CA, CB1, and CNSC of DG (*p* < 0.01) and CB1 of CM (*p* < 0.01), whereas it decreased for CB2 of CAR (*p* < 0.01), CHO, CB2, and CC of DG (*p* < 0.01), CHO, CA, CB1, CB2 and CNSC of RM (*p* < 0.05).

#### 3.3.2. Protein Composition

As shown in Table 4, the PB2 and PC of CAR increased (*p* < 0.05) by 88.5%, 32.06%, respectively, and decreased for PB3 (*p* < 0.01). The PB2 of DG increased from 8.33% to 11.65% (*p* < 0.01), whereas decreased in PB3 and PC (*p* < 0.01). Steam explosion treatment decreased PA, PB1, and PB2 of CM (*p* < 0.05), whereas it increased PB3 (*p* < 0.01). Moreover, PA and PB1 of PSR decreased (*p* < 0.05), as well as PB2 and PB3 of RM (*p* < 0.05), whereas PA and PB1 of RM increased (*p* < 0.01).

## 4. Discussion

Chang et al. [19] reported that the steam explosion treatment reduced the content of cellulose, hemicellulose and lignin of corn stover by 50.45%, 8.47% and 36.65%, respectively. The decrease in NDF, ADF and ADL in this study indicates degradation of hemicellulose, cellulose and lignin during steam-explosion processing. Moreover, the content of NDF showed a larger decrease than ADF, possibly due to the differences in rates of degradation and contents of hemicellulose and cellulose. The effect of steam explosion treatment on wheat straw showed that the degradation rate of hemicellulose was the highest (about 70%), and that of cellulose and lignin was only about 15% [20]. Furthermore, the low pressure and short retention time during the steam-explosion treatment may result in incomplete degradation of these components [21], used to explain the lack of reduction in NDF and ADF with some samples and the degradation rate of cellulose, hemicellulose and lignin increased concomitantly with an increase of steam explosion pressure [22]. Other studies have reported that the increase of ADL varies in different steam-exploded materials [22,23], similar to our result. Moreover, the increase in ADL might be caused by the degradation of hemicellulose sugars and the formation of degradation products [24], which were converted to acidic insoluble pseudo-lignin later [25]. Ballesteros et al. [22] reported that condensation and repolymerization reactions slightly increased the content of acid-insoluble lignin. To increase the degradation of lignocellulose, the steam explosion pressure and retention time need adjustment. Raw feedstuffs were high in fiber and difficult to digest, resulting in low disappearance. As pre-treatment, steam explosion makes feedstuffs more accessible to cellulase attack [26] and increase the degradation rate of cellulose, hemicellulose, lignin [18], and dry matter digestibility. This study only focused on the degradation rate of IVDMD of feedstuffs cultured in artificial rumen liquor. In the future, the digestibility of neutral detergent fiber and organic matter can also be determined. Moreover, animal feeding experiments should be performed, to more accurately explore the nutritive value of feedstuffs.

In addition to the influence of steam explosion parameters such as pressure and retention time, the above experimental results might also be caused by inadequate moisture content or unsuitable particle size [27], even inhibits formation of digestion products during steam explosion treatment. The study of different steam explosion pressures and different wheat straw to water ratios (%) suggested that a pressure of 0.8 MPa and a ratio of wheat straw to water of 1:20 are suitable for destroying ADL [20]. Ballesteros et al. [28] reported that a particle size of 8–12 mm resulted in the best steam explosion effect for herbage, and if the particle size of material was too small, this was not only sub-optimal for the effects of steam explosion, but also led to increased energy consumption. Ma et al. [29] found that the steam explosion of barley straw at 210 °C for 15 min produced inhibitors of enzymatic hydrolysis, resulting in a cellulose conversion 25% lower than that for the control.

Chaji et al. [30] studied the predicted ME of sugarcane pith treated with steam explosion treatment by in vitro gas production and the results showed that ME of a sample treated with 180–210°C for 3 min increased by 44.43% when compared with untreated ones. In this study, increased TDN and energy values may result from increased nutritive value, and those without increased possibly due to unsuitable steam explosion parameters and even the production of inhibit digestion. In the future, additional steam explosion parameters (e.g., pressure, retention time) and material conditions (moisture, particle size) need to be explored, then to determine the optimal steam explosion treatment for specific feedstuffs.

The CNCPS divided the carbohydrate content into four components according to the degradation rate of feedstuffs in rumen [18]. CA is sugar and it is rapidly degraded and CB1 is starch, which is degraded slower. Currently, increased CA of CAR, DG and PSR, as well as CB1 of DGmainly because of the greater degradation of hemicellulose and cellulose by the steam explosion treatment [20], which is also consistent with increased IVDMD. 

The protein components of CNCPS were closely related to the degradation and utilization of feedstuff in the rumen. All PA, most PB1, PB2 and a small amount of PB3 are rumen-degradable proteins, all PC, a small amount of PB1, PB2 and a large amount of PB3 are rumen-undegradable proteins [18]. The increase of PA, PB1, PB2 and IVDMD is possibly due to partial protein degradation by steam explosion treatment. Therefore, steam explosion treatment improved feedstuff stability and increased the amount of undegradable protein.

## 5. Conclusions

The results of this study show that steam-explosion treatment improved the nutritive value and IVDMD of CAR, DG, RM and PSR. Therefore, steam-explosion treatment offers the potential to improve the suitability of byproduct feedstuffs as ruminant feed. There was no effect of steam explosion treatment on CM, indicating that some feedstuffs do not react to steam-explosion treatment and should not be treated in this manner. Further studies are requried to explore more suitable steam-explosion parameters for more specific byproduct feedstuffs, including moisture content, particle size of feedstuffs and pressure retention time of the steam explosion.

## Figures and Tables

**Table 1:** Effect of steam explosion treatment on the chemical composition and in vitro digestibility of five byproduct feedstuffs (DM basis, expressed as %).

Item ^1^	Treatment ^2^	DM	NDF	ADF	ADL	CP	EE	Ash	48 h IVDMD
CAR	Raw	96.42	68.51	54.27	12.71	13.31	1.86	15.87	26.17
	Steam explosion	99.62	58.48	52.18	15.46	13.82	2.33	15.86	34.56
	SEM ^3^	0.107	0.107	0.126	0.116	0.164	0.092	0.154	0.169
	*p*-Value	<0.001	<0.001	<0.001	0.003	0.004	0.103	0.981	<0.001
DG	Raw	95.44	54.99	40.49	13.66	15.30	3.83	10.46	30.82
	Steam explosion	95.48	44.52	36.27	11.36	16.84	4.34	10.86	40.19
	SEM	0.093	0.32	0.186	0.178	0.053	0.127	0.152	0.198
	*p*-Value	0.777	<0.001	<0.001	0.001	<0.001	0.002	0.186	<0.001
CM	Raw	89.32	40.76	27.63	9.66	42.46	0.85	9.83	33.60
	Steam explosion	94.19	42.88	26.00	8.90	41.14	0.79	10.4	36.94
	SEM	0.045	0.518	0.218	0.136	0.233	0.046	0.24	0.28
	*p*-Value	<0.001	0.173	0.007	0.039	0.07	0.454	0.05	<0.001
RM	Raw	90.99	65.70	41.62	17.51	43.22	0.59	7.16	28.96
	Steam explosion	94.90	64.62	41.11	17.9	43.48	0.83	8.79	33.81
	SEM	0.129	0.03	0.178	0.105	0.05	0.035	0.013	0.176
	*p*-Value	<0.001	0.002	0.029	0.19	0.092	0.049	<0.001	0.001
PSR	Raw	92.04	20.72	17.09	2.42	10.03	1.00	8.44	45.91
	Steam explosion	98.10	19.62	17.81	2.50	9.82	0.60	5.00	49.96
	SEM	0.078	0.207	0.207	0.112	0.097	0.031	0.132	0.156
	*p*-Value	<0.001	0.065	0.035	0.677	0.015	0.018	0.002	0.002

^1^ CAR: Cassava alcohol residues; DG: Distillers’ grains; CM: Cottonseed meal; RM: Rapeseed meal; PSR: Potato starchy residue. ^2^ DM: Dry matter; NDF: Neutral detergent fiber; ADF: Acid detergent fiber; ADL: Acid detergent fiber; CP: Crude protein; EE: Ether extract; IVDMD: *In vitro* dry matter digestibility. ^3^ SEM: standard error of the mean.

**Table 2:** Effect of steam explosion treatment on the energy values of five byproduct feedstuffs (DM basis, expressed as %).

Item ^1^	Treatment ^2^	TDN	DE	ME	NEm	NEg
CAR	Raw	17.66	4.02	3.29	2.27	1.58
	Steam explosion	26.86	6.11	5.00	3.60	2.63
	SEM ^3^	0.074	0.018	0.013	0.011	0.008
	*p*-Value	<0.001	<0.001	<0.001	<0.001	<0.001
DG	Raw	34.39	7.82	6.41	4.76	3.52
	Steam explosion	42.93	9.76	8.00	6.39	4.82
	SEM	0.195	0.045	0.036	0.038	0.032
	*p*-Value	<0.001	0.001	<0.001	<0.001	0.001
CM	Raw	44.75	10.17	8.34	6.80	5.17
	Steam explosion	43.83	9.96	8.17	6.59	4.99
	SEM	0.60	0.137	0.111	0.135	0.112
	*p*-Value	0.305	0.514	0.515	0.515	0.504
RM	Raw	35.17	7.99	6.55	4.89	3.62
	Steam explosion	35.67	8.11	6.65	4.97	3.68
	SEM	0.033	0.008	0.006	0.005	0.006
	*p*-Value	0.012	0.014	0.015	0.015	0.031
PSR	Raw	64.11	14.57	11.95	13.46	11.29
	Steam explosion	65.87	14.97	12.28	14.33	12.13
	SEM	0.234	0.025	0.044	0.114	0.111
	*p*-Value	0.062	0.053	0.025	0.021	0.022

^1^ CAR: Cassava alcohol residues; DG: Distillers’ grains; CM: Cottonseed meal; RM: Rapeseed meal; PSR: Potato starchy residue. ^2^ TDN: Total digestible nutrients; DE: Digestible energy; ME: Metabolic energy; NEm: Net energy for maintenance; NEg: Net energy for gain. ^3^ SEM: Standard error of the mean.

**Table 3:** Effect of steam explosion on the carbohydrate composition of five byproduct feedstuffs (DM basis, expressed as %).

Item ^1^	Treatment ^2^	CHO	CA	CB1	CB2	CC	CNSC
CAR	Raw	68.96	8.34	1.22	28.91	30.49	9.56
	Steam explosion	67.98	16.02	1.32	13.54	37.11	17.34
	SEM ^3^	0.399	0.423	0.018	0.284	0.279	0.406
	*p*-Value	0.135	0.002	0.104	0.001	0.003	0.002
DG	Raw	70.42	15.45	4.64	17.6	32.80	20.02
	Steam explosion	67.96	20.28	5.69	14.72	27.26	25.97
	SEM	0.325	0.57	0.028	0.234	0.425	0.522
	*p*-Value	0.007	0.002	0.002	0.002	0.001	0.01
CM	Raw	46.86	9.30	0.47	13.91	23.18	9.77
	Steam explosion	47.66	9.59	0.72	16.00	21.35	10.31
	SEM	0.511	0.713	0.005	0.815	0.329	0.711
	*p*-Value	0.231	0.843	<0.001	0.116	0.039	0.715
RM	Raw	49.02	0.26	0.21	6.39	42.02	0.52
	Steam explosion	46.89	0.17	0.14	3.62	42.96	0.30
	SEM	0.098	0.022	0.005	0.254	0.253	0.045
	*p*-Value	0.005	0.011	0.017	0.03	0.189	0.048
PSR	Raw	80.53	50.77	11.46	12.49	5.80	62.23
	Steam explosion	84.59	52.7	12.49	13.39	6.01	65.18
	SEM	0.254	0.538	0.133	0.146	0.269	0.404
	*p*-Value	0.002	0.01	0.057	0.074	0.66	0.019

^1^ CAR: Cassava alcohol residues; DG: Distillers’ grains; CM: Cottonseed meal; RM: Rapeseed meal; PSR: Potato starchy residue. ^2^ CHO: Carbohydrate; CA: Sugar; CB1: Starch; CB2: Available cell wall; CC: Unavailable cell wall; CNSC: Non-structural carbohydrate. ^3^ SEM: Standard error of the mean.

**Table 4:** Effect of steam explosion on the protein composition of five byproduct feedstuffs (DM basis, expressed as %).

Item ^1^	Treatment ^2^	PA	PB1	PB2	PB3	PC
CAR	Raw	2.29	0.20	2.00	4.16	4.96
	Steam explosion	2.37	0.04	3.77	1.28	6.55
	SEM ^3^	0.09	0.012	0.032	0.077	0.155
	*p*-Value	0.074	0.007	<0.001	0.002	0.011
DG	Raw	2.72	0.33	8.33	1.66	3.06
	Steam explosion	2.51	0.24	11.65	0.87	1.72
	SEM	0.101	0.012	0.193	0.052	0.039
	*p*-Value	0.14	0.046	0.005	<0.001	<0.001
CM	Raw	6.77	1.41	33.73	1.75	1.65
	Steam explosion	6.38	1.18	29.44	3.61	1.98
	SEM	0.09	0.093	0.107	0.092	0.114
	*p*-Value	0.018	0.003	<0.001	0.001	0.098
RM	Raw	0.94	0.10	13.21	12.65	17.65
	Steam explosion	8.77	1.62	5.58	11.8	17.53
	SEM	0.038	0.019	0.039	0.104	0.118
	*p*-Value	<0.001	<0.001	<0.001	0.013	0.068
PSR	Raw	8.39	1.3	0.72	0.65	0.64
	Steam explosion	1.17	0.20	7.47	0.51	0.75
	SEM	0.047	0.09	0.064	0.07	0.088
	*p*-Value	<0.001	0.022	<0.001	0.199	0.184

^1^ CAR: Cassava alcohol residues; DG: Distillers’ grains; CM: Cottonseed meal; RM: Rapeseed meal; PSR: Potato starchy residue. ^2^ PA: Non-protein nitrogen; PB1: Rapid degraded protein; PB2: Intermediately degraded protein; PB3: Slowly degraded protein; PC: Bound protein. ^3^ SEM: Standard error of the mean.

## References

[1] Herrero M., Thornton P.K. (2013). Livestock and global change: Emerging issues for sustainable food systems. Proc. Natl. Acad. Sci. USA.

[2] Ajila C.M., Brar S.K., Verma M., Tyagi R.D., Godbout S., Valéro J.R. (2012). Bio-processing of agro-byproducts to animal feed. Crit. Rev. Biotechnol..

[3] Liang J., Lu Q., Lerner R., Sun X., Zeng H., Liu Y. (2011). Agricultural Wastes. Water Environ. Res..

[4] Naeem M., Rajput N., Zhang L.L., Zhuang S., Yan R., Wang T. (2014). Utilization of Steam Treated Agricultural by–Product as Ruminant Feed. Pak. J. Agric. Sci..

[5] Loor J., Elolimy A., McCann J. (2016). Dietary impacts on rumen microbiota in beef and dairy production. Anim. Front..

[6] Dong L., Cao G., Wu J., Liu B., Xing D., Zhao L., Zhou C., Feng L., Ren N. (2019). High-solid pretreatment of rice straw at cold temperature using NaOH/Urea for enhanced enzymatic conversion and hydrogen production. Bioresour. Technol..

[7] Pandey A. (1992). Recent process developments in solid-state fermentation. Process. Biochem..

[8] Li B., Yang W., Nie Y., Kang F., Goff H.D., Cui S.W. (2019). Effect of steam explosion on dietary fiber, polysaccharide, protein and physicochemical properties of okara. Food Hydrocoll..

[9] Jacquet N., Maniet G., Vanderghem C., Delvigne F., Richel A. (2015). Application of Steam Explosion as Pretreatment on Lignocellulosic Material: A Review. Ind. Eng. Chem. Res..

[10] Holtzapple M.T., Humphrey A.E., Taylor J.D. (1989). Energy requirements for the size reduction of poplar and aspen wood. Biotechnol. Bioeng..

[11] Association of Official Analytical Chemists (AOAC) (2000). Official Methods of Analysis.

[12] Van Soest P., Robertson J., Lewis B. (1991). Methods for Dietary Fiber, Neutral Detergent Fiber, and Nonstarch Polysaccharides in Relation to Animal Nutrition. J. Dairy Sci..

[13] Robertson J.B., van Soest P.J., James W.P.T., Theander O. (1981). The detergent system of analysis and its application to human foods. The Analysis of Dietary Fiber in Food.

[14] Menke K.H., Raab L., Salewski A., Steingass H., Fritz D., Schneider W. (1979). The estimation of the digestibility and metabolizable energy content of ruminant feedingstuffs from the gas production when they are incubated with rumen liquor in vitro. J. Agric. Sci..

[15] NRC (National Research Council) (2016). Nutrient Requirements of Beef Cattle.

[16] Weiss W. (1993). Predicting Energy Values of Feeds. J. Dairy Sci..

[17] Licitra G., Hernandez T., Van Soest P. (1996). Standardization of procedures for nitrogen fractionation of ruminant feeds. Anim. Feed. Sci. Technol..

[18] Sniffen C.J., O’Connor J.D., Van Soest P.J., Fox D.G., Russell J.B. (1992). A net carbohydrate and protein system for evaluating cattle diets: II. Carbohydrate and protein availability. J. Anim. Sci..

[19] Chang J., Yin Q.Q., Ren T.B., Song A.D., Zuo R.Y., Guo H.W. (2012). Effect of Steam Explosion Pretreatment and Microbial Fermentation on Degradation of Corn Straw. Advanced Materials Research.

[20] Zhang L.H., Li D., Wang L.J., Wang T.P., Zhang L., Chen X.D., Mao Z.H. (2008). Effect of steam explosion on biodegradation of lignin in wheat straw. Bioresour. Technol..

[21] Liu J., Ørskov E. (2000). Cellulase treatment of untreated and steam pre-treated rice straw—Effect on in vitro fermentation characteristics. Anim. Feed. Sci. Technol..

[22] Ballesteros M., Oliva J., Negro M.J., Manzanares P., Ballesteros I. (2004). Ethanol from lignocellulosic materials by a simultaneous saccharification and fermentation process (SFS) with *Kluyveromyces marxianus* CECT 10875. Process. Biochem..

[23] Vivekanand V., Olsen E.F., Eijsink V.G., Horn S.J. (2013). Effect of different steam explosion conditions on methane potential and enzymatic saccharification of birch. Bioresour. Technol..

[24] Nelson D.A., Hallen R.T., Theander O. (1988). Formation of Aromatic–Compounds from Carbohydrates—Reaction of Xylose, Glucose, and Glucuronic—Acid in Acidic Solution at 300—Degrees—C. ACS Symp. Ser..

[25] Sannigrahi P., Kim D.H., Jung S., Ragauskas A. (2011). Pseudo-lignin and pretreatment chemistry. Energy Environ. Sci..

[26] Neves M.A.D., Kimura T., Shimizu N., Nakajima M. (2007). State of the Art and Future Trends of Bioethanol Production. J. Food Process. Eng..

[27] Cara C., Ruiz E., Ballesteros I., Negro M.J., Castro E., Galiano E.C. (2006). Enhanced enzymatic hydrolysis of olive tree wood by steam explosion and alkaline peroxide delignification. Process. Biochem..

[28] Ballesteros I., Oliva J., Negro M.J., Manzanares P., Ballesteros M. (2002). Enzymic hydrolysis of steam exploded herbaceous agricultural waste (*Brassica carinata*) at different particule sizes. Process. Biochem..

[29] García-Aparicio M.P., Ballesteros I., Gonzalez A., Oliva J., Ballesteros M., Negro M.J. (2006). Effect of Inhibitors Released During Steam-Explosion Pretreatment of Barley Straw on Enzymatic Hydrolysis. Appl. Biochem. Biotechnol..

[30] Chaji M., Mohammadab T., Mamouei M., Tabatabaei S. (2010). The Efect of Processing with High Steam and Sodium Hydroxide on Nutritive Value of Sugarcane Pith by in vitro Gas Production. J. Anim. Veter.-Adv..

